# Supplementary material for: Structural determinants of voltage-gating properties in calcium channels
Source: eLife. 2021 Mar 30;10:e64087. doi: 10.7554/eLife.64087 (PMC8099428; doi:10.7554/eLife.64087)
Supplement: Supplementary file 1. [file elife-64087-supp1.docx]

**Supplementary file 1. Mean values and confidence intervals for probabilities and mean first passage times calculated from the Bayesian MSM, the confidence intervals are calculated at a confidence level of 95%.**

| **VSD I WT** | mean / µs | lower limit | upper limit |
| --- | --- | --- | --- |
| mfpt(Resting 3-Activated) | 260 | 130 | 350 |
| mfpt(Activated-Resting 3) | 558 | 300 | 710 |
| mfpt(Resting 2-Resting 3) | 560 | 320 | 760 |
| mfpt(Resting 3-Resting 2) | 2126 | 1800 | 2600 |
| mfpt(Resting 1-Resting 2) | 611 | 380 | 850 |
| mfpt(Resting 2-Resting 1) | 2354 | 2000 | 2650 |

| **VSD IVe** | mean / µs | lower limit | upper limit |
| --- | --- | --- | --- |
| mfpt(Resting 3-Activated) | 0.9 | 0.5 | 1.5 |
| mfpt(Activated-Resting 3) | 1.3 | 0.7 | 3.6 |
| mfpt(Resting 2-Resting 3) | 0.5 | 0.3 | 1.3 |
| mfpt(Resting 3-Resting 2) | 1.1 | 0.8 | 2.6 |
| mfpt(Resting 1-Resting 2) | 0.8 | 0.4 | 2.0 |
| mfpt(Resting 2-Resting 1) | 1.2 | 0.9 | 1.7 |

| **VSD IVa** | mean / µs | lower limit | upper limit |
| --- | --- | --- | --- |
| mfpt(Resting 3-Activated) | 3.0 | 2.0 | 4.7 |
| mfpt(Activated-Resting 3) | 6.0 | 4.5 | 8.0 |
| mfpt(Resting 2-Resting 3) | 0.6 | 0.3 | 1.2 |
| mfpt(Resting 3-Resting 2) | 1.0 | 0.7 | 1.9 |
| mfpt(Resting 1-Resting 2) | 3.0 | 2.1 | 4.1 |
| mfpt(Resting 2-Resting 1) | 6.0 | 4.0 | 7.5 |

| **VSD I E87A/E90A** | mean / µs | lower limit | upper limit |
| --- | --- | --- | --- |
| mfpt(Resting 3-Activated) | 8.0 | 6.0 | 11.0 |
| mfpt(Activated-Resting 3) | 13.0 | 10.5 | 17.0 |
| mfpt(Resting 1/2-Resting 3) | 18.0 | 15.5 | 20.0 |
| mfpt(Resting 3-Resting 1/2) | 14.0 | 12.5 | 16.0 |

| **VSD I E87A** | mean / µs | lower limit | upper limit |
| --- | --- | --- | --- |
| mfpt(Resting 3-Activated) | 142 | 90 | 200 |
| mfpt(Activated-Resting 3) | 221 | 180 | 310 |
| mfpt(Resting 2-Resting 3) | 484 | 375 | 680 |
| mfpt(Resting 3-Resting 2) | 271 | 195 | 360 |
| mfpt(Resting 1-Resting 2) | 157 | 100 | 220 |
| mfpt(Resting 2-Resting 1) | 430 | 280 | 570 |

| **VSD I E90A** | mean / µs | lower limit | upper limit |
| --- | --- | --- | --- |
| mfpt(Resting 3-Activated) | 5.0 | 3.0 | 7.5 |
| mfpt(Activated-Resting 3) | 8.0 | 5.5 | 10.0 |
| mfpt(Resting 1/2-Resting 3) | 14.0 | 11.8 | 17.0 |
| mfpt(Resting 3-Resting 1/2) | 10.0 | 8.7 | 12.4 |
