# Supplementary material for: Structural determinants of voltage-gating properties in calcium channels
Source: eLife. 2021 Mar 30;10:e64087. doi: 10.7554/eLife.64087 (PMC8099428; doi:10.7554/eLife.64087)
Supplement: Supplementary file 2. [file elife-64087-supp2.docx]

**Supplementary file 2. Current properties of E87A/E90A, E87A, E90A and WT controls**

| Parameters |  | Ca_V_1.1e WT |  | Ca_V_1.1e E87A/E90A |  | *p*-value |  | Ca_V_1.1e WT |  | Ca_V_1.1e E87A |  | *p*-value |  | Ca_V_1.1e WT |  | Ca_V_1.1a E90A |  | *p*-value |
| --- | --- | --- | --- | --- | --- | --- | --- | --- | --- | --- | --- | --- | --- | --- | --- | --- | --- | --- |
|  |  |  |  |  |  |  |  |  |  |  |  |  |  |  |  |  |  |  |
| *I*_peak_ (pA/pF) |  | -6.7±1.6 |  | -3.0±0.5 |  | 0.03***** |  | -16.6±2.5 |  | -10.6±2.1 |  | 0.08 |  | -9.4±2.0 |  | -4.6±0.6 |  | 0.04***** |
| *G*_max_ (nS/nF) |  | 132.0±25.7 |  | 87.1±16.7 |  | 0.17 |  | 288.5±38.4 |  | 263.5±51.5 |  | 0.71 |  | 182.8±31.5 |  | 101.2±17.5 |  | 0.04***** |
| *V*_1/2_ (mV) |  | 10.8±1.5 |  | 29.0±1.6 |  | ******** |  | 7.7±0.9 |  | 20.0±1.5 |  | **** |  | 9.0±1.8 |  | 16.7±1.7 |  | 0.008****** |
| *k*_act_ (mV) |  | 4.8±0.4 |  | 8.3±0.8 |  | 0.004****** |  | 4.0±0.4 |  | 5.8±0.4 |  | 0.003****** |  | 4.6±0.4 |  | 6.5±0.3 |  | 0.005****** |
| *V*_rev_ (mV) |  | 75.4±2.4 |  | 88.4±4.9 |  | 0.047***** |  | 77.4±1.9 |  | 75.6±1.6 |  | 0.47 |  | 74.1±2.1 |  | 85.8±6.1 |  | 0.09 |
| Time to peak (ms) |  | 84.1±11.3 |  | 17.1±1.1 |  | ******** |  | 75.9±12.0 |  | 66.5±5.6 |  | 0.47 |  | 80.0±11.5 |  | 16.1±2.9 |  | ******* |
| *R*_500_ (%) |  | 41.0±6.4 |  | 31.5±6.0 |  | 0.31 |  | 46.0±4.3 |  | 36.0±5.0 |  | 0.16 |  | 42.4±4.7 |  | 29.3±5.5 |  | 0.10 |
| *n*(*n Ipeak*) |  | 7(7) |  | 8(9) |  | -- |  | 7(7) |  | 8(8) |  | -- |  | 7(7) |  | 7(7) |  | -- |
| tau slow (ms) (n) |  | 30.3±3.9 (4) |  | -- |  | -- |  | 25.0±5.6 (4) |  | 15.4±1.2 (5) |  | 0.24 |  | 49.9±20.5 (4) |  | -- |  | -- |
| A slow (pA/pF) (n) |  | 2.8±0.7 (4) |  | -- |  | -- |  | 7.6±2.1 (4) |  | 9.4±1.7 (5) |  | 0.69 |  | 5.6±0.7 (4) |  | -- |  | -- |
| tau fast (ms) (n) |  | 7.4±1.4 (4) |  | -- |  | -- |  | 8.4±2.4 (4) |  | 3.7±0.7 (5) |  | 0.20 |  | 10.7±3.2 (4) |  | -- |  | -- |
| A fast (pA/pF) (n) |  | 1.1±0.2 (4) |  | -- |  | -- |  | 4.4±2.0 (4) |  | 2.1±0.3 (5) |  | 0.09 |  | 4.7±3.3 (4) |  | -- |  | -- |
| tau mono (ms) (n) |  | 22.3±3.8 (7) |  | 4.5±0.7 (9) |  | ******* |  | 18.2±3.6 (7) |  | 15.4±2.0 (8) |  | 0.84 |  | 18.4±3.6 (7) |  | 4.1±0.4 (7) |  | 0.003****** |
| A mono (pA/pF) (n) |  | 6.1±1.7 (7) |  | 3.1±0.5 (9) |  | 0.3 |  | 15.5±2.4 (7) |  | 8.9±2.3 (8) |  | 0.08 |  | 8.6±1.7 (7) |  | 4.9±0.7 (7) |  | 0.22 |
|  |  |  |  |  |  |  |  |  |  |  |  |  |  |  |  |  |  |  |
| All data are presented as mean ± SE. P-values were calculated using the student t-test. * p<0.05, ** p<0.01, *** p<0.001, ****p<0.0001 | | | | | | | | | | | | | | | | | | |
