# Supplementary material for: Structural determinants of voltage-gating properties in calcium channels
Source: eLife. 2021 Mar 30;10:e64087. doi: 10.7554/eLife.64087 (PMC8099428; doi:10.7554/eLife.64087)
Supplement: Supplementary file 3. [file elife-64087-supp3.docx]

**Supplementary file 3: Linear Interaction Energy (LIE) calculations with the programm *cpptraj* for the two VSDs and the mutants to calculate the electrostatic interactions of the S4 helix with all other parts of the voltage sensor.**

|  | **LIE/ kcal/mol** | | | | |
| --- | --- | --- | --- | --- | --- |
|  | **VSD I WT** | **VSD I E87A/E90A** | **VSD I  E87A** | **VSD I  E90A** | **VSD  WT IVe** |
| Activated | -412 | -168 | -212 | -206 | -296 |
| Resting 3 | -414 | -12 | -126 | -32 | -234 |
| Resting 2 | -490 |  | -178 |  | -164 |
| Resting 1 | -132 | -6 | -12 | -19 | -127 |
