# Supplementary material for: Structural determinants of voltage-gating properties in calcium channels
Source: eLife. 2021 Mar 30;10:e64087. doi: 10.7554/eLife.64087 (PMC8099428; doi:10.7554/eLife.64087)
Supplement: Supplementary file 4. [file elife-64087-supp4.docx]

**Supplementary file 4: Summary table of the modelled loops in the voltage sensors with ab-initio Rosetta (Robetta).**

| **S3-S4 Loop** | **Residues** | **Length** |
| --- | --- | --- |
| **VSDI** | 140-161 | 23 |
| **VSDII** | 513-525 | 13 |
| **VSDIII** | 885-892 | 8 |
| **VSDIV** | 1205-1229 | 23 |
