# Supplementary material for: Structural determinants of voltage-gating properties in calcium channels
Source: eLife. 2021 Mar 30;10:e64087. doi: 10.7554/eLife.64087 (PMC8099428; doi:10.7554/eLife.64087)
Supplement: Supplementary file 5. [file elife-64087-supp5.docx]

**Table 5: Primers used for site-directed mutagenesis.**

| **Construct** | **PCR product** | **Forward primer** | **Reverse (complement) primer** |
| --- | --- | --- | --- |
| Ca_V_1.1e-E87A/E90A | 348 bp | TTTGTAACAGCTGCT | GAAGTACGCCAGCTTCGCCAGGCCCAGGTTCAGGGAGTTGT |
| Ca_V_1.1e-E87A/E90A | 862 bp | GGGCCTGGCGAAGCTGGCGTACTTCTTCCTCACCGTCTTCT | CGTGATCCAGCTCATGTA |
| Ca_V_1.1e-E87A | 348 bp | TTTGTAACAGCTGCT | GAAGTACTCCAGCTTCGCCAGGCCCAGGTTCAGGGAGTTGT |
| Ca_V_1.1e-E87A | 862 bp | GGGCCTGGCGAAGCTGGAGTACTTCTTCCTCACCGTCTTCT | CGTGATCCAGCTCATGTA |
| Ca_V_1.1e-E90A | 348 bp | TTTGTAACAGCTGCT | GAAGTACGCCAGCTTCTCCAGGCCCAGGTTCAGGGAGTTGT |
| Ca_V_1.1e-E90A | 862 bp | GGGCCTGGAGAAGCTGGCGTACTTCTTCCTCACCGTCTTCT | CGTGATCCAGCTCATGTA |
| *Mutated nucleotides are indicated in red.* | | | |
