# Supplementary material for: Structural determinants of voltage-gating properties in calcium channels
Source: eLife. 2021 Mar 30;10:e64087. doi: 10.7554/eLife.64087 (PMC8099428; doi:10.7554/eLife.64087)
Supplement: Supplementary file 6. [file elife-64087-supp6.docx]

**Supplementary file 6. Input Scripts**

**Supplementary file 6a. Two required scripts and flags to perform the Rosetta cyclic coordinate descent (CCD) algorithm to build unresolved loops.**

#flags_CCD

-in:file:s input_pdb

-in:file:fullatom

-loops:loop_file chainbreak_fix.loops

-nstruct 100

-loops:remodel

-loops:remodel perturb_ccd

-loops:refine refine_ccd

-use_input_sc

-out:file:fullatom

-out:path:all output_files

-out:file:scorefile chainbreak_fix.fasc

#chainbreak_fix

LOOP 140 161 0 0 1

**Supplementary file 6b.** **AMBER input protocols for setting up the MD simulations for structure refinement and production runs.**

#flags_CCD

-in:file:s input_pdb

-in:file:fullatom

-loops:loop_file chainbreak_fix.loops

-nstruct 100

-loops:remodel

-loops:remodel perturb_ccd

-loops:refine refine_ccd

-use_input_sc

-out:file:fullatom

-out:path:all output_files

-out:file:scorefile chainbreak_fix.fasc

#tleap file to generate topology and coordinate files as starting structures for minimizations, equilibrations and production runs

source leaprc.lipid14

source leaprc.protein.ff14SBonlysc

source leaprc.water.tip3p

loadAmberParams frcmod.ionsff99_tip3p

loadoff ions94.lib

loadoff atomic_ions.lib

pdb = loadPdb input_tleap.pdb

charge pdb

AMBERsetBox pdb centers

saveAmberParm pdb complex.parm7 complex.inpcrd

savePdb pdb complex.pdb

quit

**Supplementary file 6c**. **AMBER input protocols for setting up 10ns of MD simulations.**

#production input file for 10ns

pro 10ns

&cntrl

imin=0, ntx=5, irest=1,

ntc=2, ntf=2,

nstlim=5000000,

ntt=3, gamma_ln=5.0, ig=-1,

temp0=300.0,

ntpr=1000, ntwr=5000, ntwx=1000,

dt=0.002, iwrap=1,

ntb=2, ntp=2, pres0=1.0,taup=5.0, cut=8.0, ioutfm=1

/

&ewald skinnb=3.0

/

savePdb pdb complex.pdb

quit
